# The psychological impact on parents of children with pyridoxine-dependent epilepsy

**DOI:** 10.1192/j.eurpsy.2023.1538

**Published:** 2023-07-19

**Authors:** I. Boujelbene, M. Chaabane, M. Guirat, D. Ben Touhemi, S. Guidara, Y. Moalla, H. Kamoun, I. Ben Ayed

**Affiliations:** 1 Department of Medical Genetic; 2 Department of Child Psychiatry, Hedi Chaker Hospital, Sfax, Tunisia

## Abstract

**Introduction:**

Pyridoxine-dependent epilepsy (PDE) is a rare autosomal recessive disease usually associated with neonatal seizures that are sensitive to pyridoxine (vitamin B6). This disease can have a significant impact on family functioning, with significant psychological distress in parents. Post-traumatic stress disorder (PTSD), depression, and anxiety are the most common psychiatric outcomes in parents of children with PDE.

**Objectives:**

To investigate the prevalence of significant symptoms of depression, anxiety, and stress in parents of children with PDE.

**Methods:**

The study consisted of a survey of parents accompanying their children diagnosed with PDE. The diagnosis was already confirmed by objectifying a homozygous or a compound heterozygous mutation in the *ALDH7A1* gene in all siblings with heterozygous carrier parents. The Impact of Event Scale-Revised (IES-R) was used to assess parental post-traumatic stress, and the Hospital Anxiety and Depression Scale was used to screen for parental depression and anxiety.

**Results:**

Our study included eight unrelated families with one infant presenting a confirmed PDE disease. The average age of the children with epilepsy was 4.18 years (8 months to 12 years) with equal representation of both sexes.

Half of parents surveyed had depressive symptoms and about two thirds reported anxious symptomatology. These troubles are mainly related to the uncertain prognosis of the disease, even with vitamin B6 supplementation, and the high risk of recurrence in siblings, which led some parents to not have other children. A higher anxiety scores was reported in parents who claimed to have difficulties in providing the necessary vitamin supplements to their affected children on a regular basis. PTSD was diagnosed in three parents: most parents reported difficulties in dealing with stress, specifically in relation to the unpredictability of seizures and the unavailability of medical care for their child, which taxed their financial resources and made it difficult for them to perform their roles effectively.

Besides, being an autosomal recessive transmission disease, the notion of responsibility/guilt was not reported by either parent, and both parents are equally involved in the care of their child.

**Conclusions:**

A significant proportion of children’s parents with pyridoxine-dependent epilepsy are suffering from depression, anxiety, and post-traumatic stress. A deeper understanding of the clinical expressions of these troubles could help practitioners to develop prevention and intervention strategies for these parents.

**Disclosure of Interest:**

None Declared

